# Supplementary material for: Learning curve and surgical outcome of robotic assisted colorectal surgery with ERAS program
Source: Sci Rep. 2022 Nov 29;12:20566. doi: 10.1038/s41598-022-24665-w (PMC9709162; doi:10.1038/s41598-022-24665-w)
Supplement: Supplementary file 1 — Supplementary Information 1. [file 41598_2022_24665_MOESM1_ESM.zip › LSC Raw data-Ñ[▒K/110/O_ñw╝╨░O▒Kñσ.pdf]

正式摘要(電子簽章：000000206067702)

請選擇...reporter

臺中榮民總醫院

Operation Note

[redacted]

- 手術日期：2020/12/24
- 手術醫師：VS 蔣鋒帆，CR 楊曙榮，CR 江世偉，R 王文謙
- Anesthesia：GE
- Position：Lithotomy
- Aseptic agents：Soap，Betadine Alcohol，Betadine Aqua

Diagnosis

- Pre-operative diagnosis :S COLON CA WITH ECONOMIC LIMITATION(NO HARMONIC SCAPLE)
- Post-operative diagnosis : Ditto.

Surgical Indication

- Tumor resection

Operation

- 1. Laparoscopic anterior resection with end-to-end anastomosis

Finding

- 1. One 3x3 cm tumor located at distual sigmoid colon
  2. Moderate adhision around mesentery, mesocolon and colon
  3. There was no palpable liver metastasis, no visible peritoneal seeding.
  4. High ligation (+) above the left colic artery.
  5. Drain: 12 penrose in pelvis

Procedure

- Under GE, the patient was put in lithotomy position. 4 troca hole was made, the first was by open method. Pneumoperitonium was created. the Olympus Endo-EYE laparoscope was inserted from umbilical port. Abdominal exploration was done and the findings were mentioned above. The patient position was adjusted to head-down and right tilting to let small bowels move to RUQ and the pelvis was well exposured. The IMA was identified, dissected, skeletonized by Harmonic scalpel and transected by triple clips and Harmonic energy device. The IMV was done in the same way. The mobilization of S-colon was done with dissection via embryonic plane between Gerota fascia and meso-sigmoid colon. The lateral attachement of sigmoid colon was also divided. After fully mobilization of S-colon, the rectum was also dissected posteriorly in upper rectum level, Denovillier fascia anteriorly. The bilateral lateral ligment was divided. Transection of rectum at peritoneal reflection was done by two 60 mm endocutters. The speciemn was removed from periumbilical wound . The 33 mm envil was inserted in proximal colon annd take back into abdominal cavity. The end to end colorectal anastomosis was done by double stappling technique. After air-leak test, penrosce drain was placed to the pelvic from the LLQ troca. 2 ports and wound was closed in layers. Throughout the whole course of operation, patient stood quite well.
- Estimated blood loss: minimum
- 器械數量: 正確
- 紗布數量: 正確

Image

- PAINT
  - abdomen

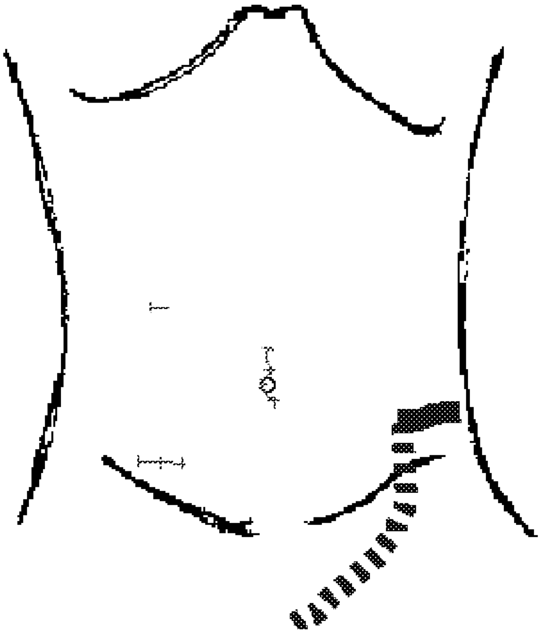

- colon & rectum

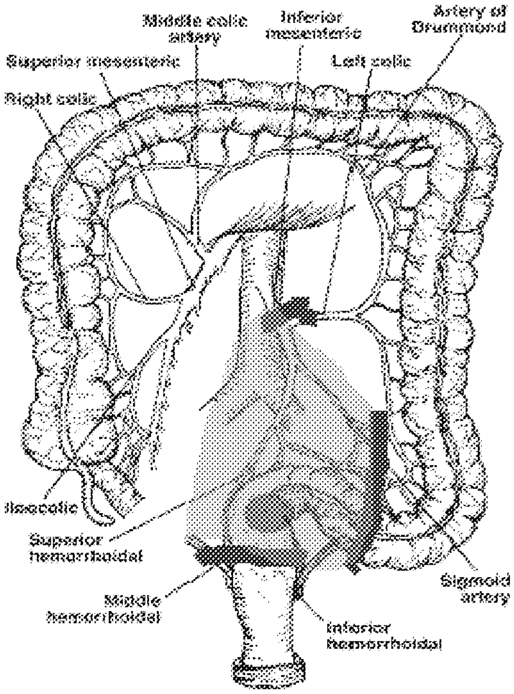

主任 2062F蔣鋒帆 / 醫師 2292G楊曙榮

正式摘要**(**電子簽章：**000000206067702)**

請選擇...reporter

doctorRank:B
